# The Role of Pharmacogenomics in Optimizing Ketamine Therapy for Post-Amputation Pain

**DOI:** 10.3390/reports8030156

**Published:** 2025-08-22

**Authors:** Alix Tappe, Emily Burzynski, Jhanvi Patel, Ithamar Cheyne, Małgorzata Mikaszewska-Sokolewicz

**Affiliations:** 1 Anesthesiology and Intensive Care Scientific Circle English Division (ANKONA ED), Medical University of Warsaw, 02-091 Warsaw, Poland; 2 Department of Anesthesiology and Intensive Care, Children’s Memorial Health Institute, 04-736 Warsaw, Poland

**Keywords:** post-amputation pain, ketamine, pharmacogenomics, NMDA receptor antagonists, CYP2B6 polymorphism, personalized pain management

## Abstract

Context and objective: Post-amputation pain (PAP) is an umbrella term that includes residual limb pain (RLP) and phantom limb pain (PLP), posing a significant challenge to recovery and quality of life after limb loss. Ketamine, an N-methyl-D-aspartate (NMDA) receptor antagonist, has gained interest for its potential to manage PAP, particularly in refractory cases. This narrative review explores the efficacy of ketamine for PAP and the emerging role of pharmacogenomics in guiding its use. Methods: A literature review of PubMed, Embase, and Cochrane databases was conducted, focusing on clinical trials, systematic reviews, and genetic influences on ketamine metabolism and response. Studies suggest that perioperative ketamine can reduce PAP severity and opioid use. However, outcomes vary, with some patients experiencing transient relief and others achieving prolonged benefit. Results: This variability may be linked to genetic differences in CYP2B6, CYP3A4/5, COMT Val158Met, SLC6A2, and KCNS1, which affect ketamine’s metabolism, efficacy and side effect profile. Understanding these pharmacogenomic factors could enable more personalized and effective ketamine therapy. Conclusion: Despite its promise, inconsistent dosing regimens and limited integration of genetic data hinder standardization. Further research into genotype-guided ketamine protocols may improve treatment outcomes and support precision analgesia in amputee care.

## 1. Introduction and Clinical Significance

PAP, affecting up to 95% of individuals who have undergone amputation [1], is a complex and challenging condition that significantly impacts the quality of life for individuals who experience limb loss. It is typically used as an umbrella term to that includes both RLP and PLP. RLP originates near the surgical site and is often associated with incision pain, scar tissue formation, or infection, typically emerging days to weeks after surgery. In contrast, PLP manifests as painful sensations in the absent limb, developing weeks to months following amputation. This chronic pain is thought to arise from cortical reorganization in the somatosensory cortex, where the brain continues to interpret signals from the amputated limb [1,2].

Among the various treatment options for PAP, ketamine has gained recognition as a compelling choice. It is a dissociative anesthetic with N-methyl-D-aspartate (NMDA) receptor antagonist properties. Over the past decades, ketamine has been the focus of multiple clinical investigations for the treatment and prevention of PAP; however, the results across studies have been inconsistent and inconclusive, reflecting variability in dosing, timing, routes of administration, and patient responses. Table 1 summarizes the regimens in the following investigations.

Nikolajsen et al. [3] investigated ketamine administration in patients with PLP and coexisting stump pain. The findings demonstrated significant relief of both stump pain and PLP during and up to 30 min after the infusion. However, in most patients (7 out of 11), the pain recurred shortly after the infusion, while in 4 patients, the pain relief lasted between 3 and 24 h.

Randomized controlled trials (RCTs) by Wilson et al. [4] and Hayes et al. [5] found that perioperative administration of ketamine may help mitigate the development of chronic PAP. Wilson et al. reported significantly lower pain scores in the group receiving ketamine, while Hayes et al. could not demonstrate a significant reduction in postoperative pain sensitization, PAP, or morphine consumption. Eichenberger et al. [6] evaluated the efficacy of ketamine and ketamine combined with calcitonin in patients who had already developed PLP after amputation. Both the ketamine-only and ketamine-plus-calcitonin groups experienced significant reductions in PLP intensity; however, only the combination therapy led to a statistically significant reduction in average and peak pain intensity 48 h post-treatment.

Low-dose intravenous (IV) ketamine has also been investigated in the context of combat-related limb injuries during the acute phase of trauma recovery [7]. Unlike other studies that primarily focus on scheduled amputation surgery or chronic pain post-surgery, a study by Polomano et al. [7] examined a patient group of 19 individuals, 11 of whom had lost their limbs primarily due to combat-related explosions, and 9 of these reported PLP. The study found a significant reduction in present pain intensity (PPI), with particularly noteworthy improvements in patients with higher baseline pain scores who did not report PLP. In a brief research report by Buvanendran et al. [8], three patients demonstrated effectiveness in treating acute pain following amputation surgery. Furthermore, the oral administration of ketamine was found to be a safe and convenient non-opioid option in PAP control, potentially enabling its use in outpatient settings.

The lack of consistent and conclusive findings across studies evaluating ketamine for PAP may, in part, reflect the underlying variability in patient responses to the drug. While differences in dosing regimens, timing, and routes of administration have contributed to these mixed outcomes, an additional and often overlooked factor is individual genetic variability. This emerging perspective suggests that some patients may be inherently more responsive to ketamine, while others may experience limited or short-lived relief.

Precision analgesia refers to tailoring pain management strategies to the individual patient, guided by genetic, clinical, and psychosocial factors, to maximize efficacy while minimizing adverse effects.

In the following section, we will explore the pharmacogenomics of ketamine to better understand how genetic factors might shape clinical outcomes and guide precision analgesia in PAP management.

## 2. Methods

This study was conducted as a narrative review aimed at synthesizing current knowledge on the role of ketamine in post-amputation pain (PAP) and examining how pharmacogenomics affects treatment outcomes. A structured yet non-systematic approach was used to gather a wide range of clinical and mechanistic evidence.

Relevant publications were identified through searches of PubMed, Embase, and the Cochrane Library from the start of the databases up to January 2025. Search terms included combinations of *“*post-amputation pain,” “phantom limb pain,” “residual limb pain,” “ketamine,” “NMDA receptor antagonists,” “pharmacogenomics,” “CYP2B6,” “CYP3A4/5,” “COMT,” “SLC6A2,” and “KCNS1.” Additional sources were identified by examining the reference lists of included articles and previous reviews.

Since this is a narrative review, formal systematic review methods (such as the PRISMA flowchart and risk of bias tools) were not used. Instead, the focus was on including studies most relevant to the clinical use of ketamine in PAP and those exploring genetic factors influencing response. Priority was given to randomized controlled trials, cohort studies, case series with translational relevance, and mechanistic and pharmacogenomic research. Publications in languages other than English, isolated case reports, and preclinical studies lacking clinical relevance were excluded.

Extracted evidence was synthesized qualitatively. Findings were organized into thematic areas such as clinical effectiveness of ketamine in PAP, mechanisms of action, pharmacogenomic factors affecting response and safety, and implications for personalized analgesia. To enhance clarity and comparison, key clinical studies and genetic associations are summarized in tables.

While our primary focus is on lower limb PAP, the current evidence base is limited. Therefore, some supporting studies from upper limb amputees, oncologic pain, or chronic pain contexts are included to provide mechanistic insights and contextual understanding.

## 3. Mechanism of Action of Ketamine

**Figure 1:**
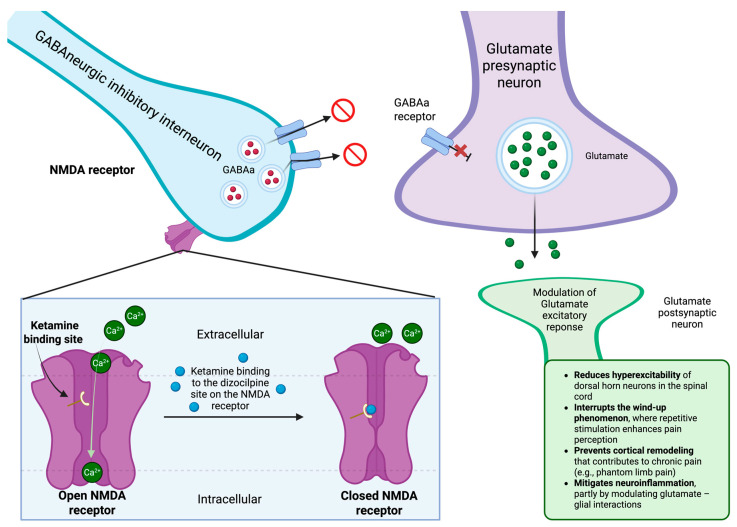
Ketamine-mediated NMDA receptor blockade and its role in modulating central sensitization in pain pathways. Illustration of how ketamine inhibits NMDA receptor activity by binding to the dizocilpine site, blocking calcium influx, resulting in GABAergic inhibition over glutaminergic neurons. (Created by the authors with the use of Biorender.) Legend: NMDA—N-methyl-D-aspartate, GABA—Gamma-Aminobutyric Acid, GABAa—Ionotropic GABA receptor, Ca^2+^—Calcium ion.

Ketamine delivers potent analgesic effects primarily by targeting the NMDA receptor, a crucial regulator of pain processing and central sensitization. As depicted in Figure 1, by binding to the NMDA receptor, it inhibits calcium influx and reduces glutamate-mediated excitatory signaling, effectively mitigating the amplification of pain signals [9]. This mechanism underlies ketamine’s anti-nociceptive, anti-hyperalgesic, and anti-neuropathic properties, making it particularly effective in managing perioperative pain, opioid-resistant pain, and neuropathic conditions.

Unlike traditional anesthetics, ketamine offers pain relief at sub-anesthetic doses, enabling analgesia without causing complete unconsciousness [10]. PAP results from the sudden loss of sensory input, leading to cortical reorganization in the somatosensory cortex and contributing to the perception of pain in the missing limb [11]. Additionally, persistent peripheral nerve activity and heightened excitability of the dorsal horn lead to prolonged NMDA receptor activation, intensifying pain sensitization and increasing the risk of a chronic pain disorder [12]. By blocking NMDA receptors, ketamine interrupts recurrent pain signaling, preventing the “wind-up” phenomenon—where persistent nerve stimulation enhances pain perception and promotes chronic pain development. Simultaneously, it reduces hyperexcitability in the spinal cord and suppresses maladaptive central nervous system changes [13]. Beyond its potent NMDA receptor blockade, ketamine interacts with opioid, monoaminergic, nicotinic, and muscarinic receptors, influencing various pain pathways, though these effects are less pronounced than its NMDA receptor antagonism [10]. Additionally, ketamine disrupts glial activation, thereby reducing neuroinflammation, an important factor in chronic pain conditions like PAP. By modulating neuroinflammatory responses and preventing central sensitization, ketamine helps avert the progression from acute postoperative pain to chronic PLP [14]. However, its analgesic efficacy depends on active NMDA receptor signaling; thus, ketamine requires ongoing nociceptive activity to exert its effects. This characteristic explains why ketamine is more effective in chronic and neuropathic pain conditions rather than in acute or preemptive analgesia [13]. Frequent indications for ketamine in pain management include cancer pain, fibromyalgia, and opioid-resistant pain [10,14]. Due to the complexity of PAP, ketamine’s multifaceted mechanism offers a promising approach to managing PLP and improving patient outcomes.

In addition to NMDA receptor antagonism, ketamine modulates chronic pain in other ways. Recent mechanistic insights highlight ketamine’s impact on the AMPA receptor, promoting BDNF release and TrkB activation, which supports synaptic plasticity and reversal of central sensitization—key processes in chronic pain resolution [15]. Ketamine also exerts anti-inflammatory effects within the CNS, including suppression of microglial activation and reduction of pro-inflammatory cytokines, which may contribute to its long-term analgesic efficacy in neuropathic pain [16]. Ketamine may attenuate aberrant cortical-thalamic activity, a mechanism implicated in PLP and maladaptive cortical plasticity, thereby restoring more physiologic pain signaling patterns [17].

Ketamine is a racemic mixture composed of two enantiomers, S-(+)-ketamine and R-(–)-ketamine, each with unique pharmacodynamic and molecular properties.

S-ketamine is a more potent NMDA receptor antagonist and is primarily responsible for ketamine’s dissociative and anesthetic properties, which explains its use in the FDA-approved intranasal formulation for treatment-resistant depression [18]. In contrast, R-ketamine, while less potent as an NMDA blockade, has shown superior efficacy in enhancing mTOR-dependent synaptogenesis, stimulating BDNF-TrkB signaling, and sustaining long-term antidepressant and analgesic effects in preclinical models [19]. Both S and R enantiomers attenuate microglial activation and pro-inflammatory cytokine expression [20].

## 4. Pharmacogenomics of Ketamine: Genetic Determinants of Response

Individual genetic variations likely contribute to the range of effectiveness and adverse effects of ketamine for PAP. Key pharmacogenomic factors include CYP2B6 and CYP3A4/5 in ketamine metabolism, brain-derived neurotrophic factor (BDNF), COMT in pain modulation, and SLC6A2 in sympathetic response. Integrating this genetic data into treatment plans is imperative for providing optimized and personalized care to those experiencing PAP.

### 4.1. CYP2B6 Alleles

The *6 variant is common in many populations, especially of African descent, and causes reduced CYP2B6 expression and activity [21]. Carriers have significantly slower ketamine clearance [22]. Recent studies confirmed that the *6 allele leads to up to 89% reduced ketamine clearance, resulting in higher plasma levels and increased incidence of dissociative symptoms in *6 homozygotes [23,24]. In chronic pain patients receiving ketamine infusions, *6 heterozygotes had approximately 40 percent of normal clearance, and 6*/6* homozygotes only about a 30 percent clearance [22]. Consequently, *6 carriers accumulate higher plasma ketamine levels (higher ketamine/norketamine ratio) predisposing them to exaggerated drug effects [22]. This variant has been linked to a higher incidence of dissociative adverse effects during ketamine therapy [22,25].

Recent mechanistic insights into ketamine metabolism have revealed that CYP2B6 polymorphisms, particularly *6 and 34 alleles, not only reduce metabolic clearance but also alter the enzymatic pathway through which ketamine is processed. A 2024 structural and biochemical study demonstrated that ketamine can induce peroxide shunt activity even in wild-type CYP2B6 enzymes, but this effect is significantly amplified in variant forms of the enzyme such as CYP2B634 [26]. The peroxide shunt represents an uncoupled metabolic pathway where electrons are diverted from normal substrate oxidation to produce reactive oxygen species (ROS) like hydrogen peroxide (H_2_O_2_). This altered catalysis may have clinical implications beyond pharmacokinetics: increased H_2_O_2_ production may contribute to oxidative stress, potentially exacerbating neurotoxic or psychotropic side effects during ketamine administration [26]. This variant-specific uncoupling could modify the ratio of ketamine to its active and inactive metabolites, thereby influencing both its analgesic duration and intensity of dissociative symptoms. These findings support a growing recognition that CYP2B6 genotyping not only predicts clearance rates but may also inform the safety profile and therapeutic efficacy of ketamine via oxidative balance and metabolite distribution [26,27]. Beyond genetic variants, recent research shows that ketamine metabolism via CYP2B6 is also significantly influenced by non-genetic factors such as liver function, drug interactions, and alcohol use, which further complicates individualized dosing [28].

### 4.2. Brain-Derived Neurotrophic Factor (BDNF)

BDNF plays a critical role in neuroplasticity, synaptic modulation, and recovery from injury—processes that are central to both chronic pain and ketamine’s mechanism of action. The Val66Met polymorphism, a common single-nucleotide polymorphism in the BDNF gene, leads to reduced activity-dependent BDNF secretion and impaired long-term potentiation. This polymorphism has been associated with diminished clinical response to ketamine in the treatment of depression [25,29]. Although the role of BDNF Val66Met in pain remains less defined, impaired neuroplasticity could theoretically contribute to poor central sensitization reversal, making Met carriers less responsive to ketamine’s analgesic effects in conditions like PLP [30]. A 2018 study by Hu et al. examined the interaction between BDNF Val66Met and CYP2B6 polymorphisms in patients treated with ketamine for depression. While not specific to pain, the study highlighted significant gene–gene interactions affecting both efficacy and tolerability [31]. These findings suggest that BDNF and COMT variants may act as genetic modifiers, influencing ketamine’s therapeutic window and risk–benefit ratio in neuropathic pain states such as PLP.

### 4.3. CYP3A4/5

CYP3A4 is highly abundant in the liver and gut, and it also metabolizes ketamine [21]. Although no common null variants exist, CYP3A4 expression varies (e.g., 22 allele reduces activity modestly). CYP3A5, often expressed in individuals of African or Asian ancestry (CYP3A51 allele), can supplement metabolism [21]. Importantly, CYP3A4 may dominate ketamine metabolism under certain conditions: for example, oral ketamine undergoes extensive first-pass metabolism by intestinal/hepatic CYP3A4, yielding high norketamine levels [21]. Changes in CYP3A4 activity, whether inhibited or induced, significantly alter ketamine levels. CYP3A4 inhibitors (like clarithromycin and grapefruit juice) greatly raise plasma ketamine, while inducers (like St. John’s wort) reduce levels [21]. Thus, CYP3A variability (genetic or via drug interactions) can alter ketamine’s bioavailability. By contrast, with IV dosing (bypassing the gut), CYP2B6 plays a larger role [21].

### 4.4. Catechol-O-Methyltransferase (COMT)

COMT metabolizes catecholamines (dopamine, norepinephrine) and influences pain modulation [32]. The common Val158Met polymorphism, frequently seen in Caucasian populations, reduces COMT activity approximately 3- to 4-fold (Met allele = lower activity) [31,32]. The COMT genotype does not directly change ketamine levels but affects endogenous pain pathways and NMDA receptor function. Low COMT activity (Met/Met) leads to higher synaptic catecholamines and has been associated with increased pain sensitivity in experimental and surgical pain models [33]. Clinically, COMT polymorphisms correlate with differing analgesic needs; for example, a meta-analysis found specific COMT genotypes required lower postoperative opioid doses [31]. This suggests COMT might also modulate ketamine’s analgesic effectiveness by altering baseline pain and descending inhibition. Although direct evidence in ketamine response is limited, COMT represents an important genetic factor for overall pain experience that could influence ketamine’s perceived efficacy.

### 4.5. SLC6A2 (Norepinephrine Transporter, NET)

Ketamine stimulates the sympathetic nervous system, raising heart rate and blood pressure. A polymorphism in the NET gene (rs28386840 A>T in the promoter) reduces NET expression [34]. Carriers of the T allele have impaired NE reuptake, leading to pronounced adrenergic responses. In a controlled trial, NET rs28386840 T carriers had a faster and greater increase in systolic blood pressure during low-dose ketamine infusion compared to AA homozygotes [34]. This variant was associated with more frequent moderate-to-severe hypertension during ketamine administration [25]. Thus, NET genetics can influence ketamine’s cardiovascular side effects without changing its analgesic action.

**Figure 2:**
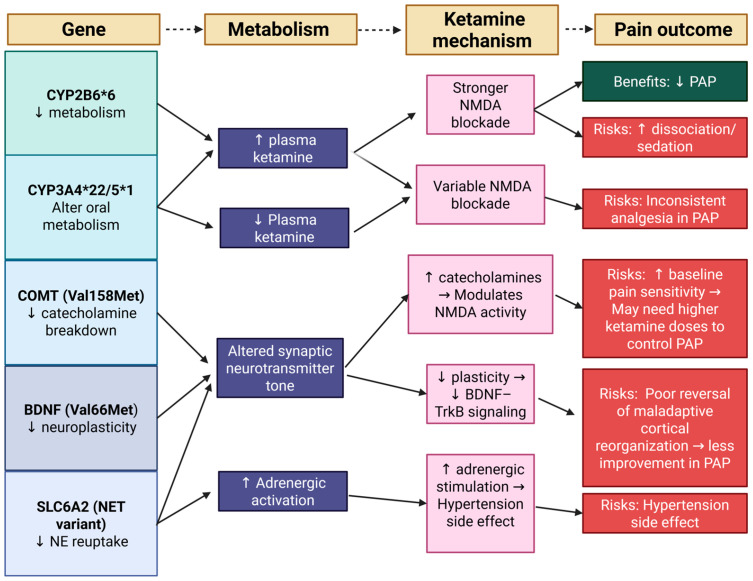
Pharmacogenomic influences on ketamine metabolism, mechanisms, and pain outcomes in PAP. This schematic illustrates how key genetic polymorphisms (CYP2B6, CYP3A4/5, COMT, BDNF, and SLC6A2) influence ketamine metabolism and pharmacodynamics. Variations in metabolism (e.g., increased or decreased plasma ketamine, altered neurotransmitter tone, or adrenergic activation) subsequently modulate ketamine’s mechanisms of action, including NMDA receptor blockade, BDNF–TrkB signaling, catecholamine modulation, and adrenergic stimulation. These pharmacogenomic differences contribute to variability in pain outcomes in PAP, influencing both therapeutic benefits (reduced PAP) and risks (e.g., dissociation, sedation, hypertension, or insufficient analgesia). (Created by the authors with the used of Biorender.)

Additional genetic factors are under investigation. Polymorphisms in NMDA receptor subunit genes (e.g., GRIN2B) have been hypothesized to influence ketamine’s psychotropic and analgesic effects, though studies so far show mixed results or no strong associations [35]. Notably, genetic predispositions to neuropathic pain may indirectly affect ketamine utility; for example, a variant in the potassium channel gene KCNS1 is associated with a higher risk of chronic neuropathic pain (including PLP) after amputation [36]. Such patients might particularly benefit from aggressive NMDA-antagonist interventions. Although these “pain risk” genes do not alter ketamine pharmacokinetics, they underscore the inter-individual differences in pain pathways that, combined with pharmacogenetics, shape clinical outcomes. Table 2 summarizes the genes involved in the pharmacogenomics of ketamine. Figure 2 provides an integrative overview of how pharmacogenomic variants influence ketamine metabolism and mechanisms, ultimately shaping analgesic efficacy and side effect profiles in PAP.

## 5. Comparison with Other Treatment Modalities

The management of PAP remains a complex challenge, often requiring a multimodal approach that incorporates pharmacological and non-pharmacological interventions. While ketamine has emerged as a promising agent, particularly in refractory cases, a wide array of other treatments, including opioids, antidepressants, anticonvulsants, local anesthetics, and alternative NMDA receptor antagonists, continue to be employed, each with distinct mechanisms, durations of action, effectiveness, and side effect profiles. Given that pharmacogenomics may impact an individual’s response to ketamine, these additional treatment options may be more appropriate for certain patients. Table 3 provides a comparative summary of key therapeutic options for PAP, highlighting their mechanisms of action, clinical utility, safety considerations, and current limitations.

## 6. Impact of Pharmacogenomics on Ketamine’s Safety Profile in Post-Amputation Pain Management

Ketamine is recognized for its extensive use in pain management and treatment-resistant depression; however, its safety profile and side effects remain a critical consideration, particularly in the perioperative management of PAP. Ketamine is often employed as an alternative to opioids, with studies suggesting that doses between 0.1 to 0.3 mg/kg can optimize analgesia while reducing opioid consumption and associated risks [46]. Importantly, the method of administration influences the severity of adverse effects—slower infusion rates, such as ten-minute infusions, have been associated with fewer psychotropic reactions compared to rapid IV administration [46].

Neuropsychiatric disturbances, notably hallucinations and emergence delirium, have been frequently reported. Cardiovascular stimulation, characterized by transient hypertension and tachycardia, is another well-documented effect due to sympathetic nervous system activation. In healthy volunteers, doses of 0.1–0.5 mg/kg are sufficient to induce dissociative symptoms and psychotic-like experiences, such as palpitations, disorientation, and perceptual alterations [46].

Long-term ketamine use, primarily studied in recreational contexts but increasingly relevant in chronic pain management, has been linked to cognitive deficits, including impairments in working memory, episodic memory, and attention, especially in males [46,47].

Genetic factors increasingly appear to modulate the risk and severity of ketamine’s side effects, particularly at subanesthetic analgesic doses relevant to PAP protocols. Genetic variability in CYP2B6, the primary hepatic enzyme responsible for ketamine N-demethylation, plays a critical role in modulating both the pharmacokinetics and safety profile of ketamine. Individuals carrying loss-of-function alleles such as *6/*6 may experience elevated plasma concentrations, longer elimination half-life, and an increased risk of adverse effects, particularly neuropsychiatric and cardiovascular toxicity [48]. Patients harboring the CYP2B6*6 allele, which confers slow ketamine metabolism, demonstrate higher peak plasma ketamine levels and significantly greater dissociative and psychotomimetic symptoms [25]. These symptoms are likely the result of excessive NMDA receptor blockade due to drug accumulation. Conversely, rapid metabolizers may clear ketamine before severe psychotropic side effects develop.

Although no specific NMDA receptor subunit polymorphism (e.g., GRIN2B) has been definitively associated with ketamine-induced hallucinations [35], research remains ongoing. Additionally, the BDNF Val66Met polymorphism has been linked to altered central nervous system responses to ketamine, including a diminished antidepressant effect. However, its impact on dissociative phenomena in pain treatment remains speculative [25].

Ketamine generally poses minimal risk for respiratory depression at analgesic doses; however, sedation may become pronounced in slow metabolizers. CYP2B6*6 homozygotes and individuals with low CYP3A4 activity are at increased risk of excessive sedation due to prolonged drug clearance [21]. Age further compounds this effect, as elderly patients exhibit naturally reduced ketamine metabolism, requiring cautious dose titration.

Pharmacogenetic variation also modulates ketamine’s cardiovascular effects. The NET gene polymorphism (rs28386840) has been associated with exaggerated sympathetic responses. Carriers of the T allele demonstrate more rapid and severe increases in systolic blood pressure during ketamine infusions [34], elevating the risk of transient hypertension or tachyarrhythmias. Pre-infusion beta-blockade or dose adjustments may be advisable in genetically susceptible individuals.

## 7. Clinical Implications for Personalized Ketamine Therapy

Considering these findings, a personalized medicine approach to ketamine use in limb amputees is on the horizon. Genotype-guided dosing could become a practical strategy to maximize analgesia while minimizing harm.

Patients known to carry CYP2B6*6 (especially homozygous) might be started at lower infusion rates or given reduced doses of ketamine. Given that these patients clear ketamine slowly [22], conservative dosing can achieve adequate plasma levels without overshooting into toxicity. Genotype information would also signal the care team to be vigilant for neuropsychiatric side effects; ancillary medications (benzodiazepines, anti-psychotics) could be readied to treat emergent severe hallucinations in these high-risk individuals.

Standard doses might underperform for rapid metabolizers (e.g., those without CYP2B6*6 and who might express CYP3A5). Though not yet routine, one could envision using genotypes to identify patients who may need higher ketamine doses or additional analgesics. For example, if a patient has no CYP2B6 variants and is a CYP3A5 expresser, their ketamine clearance might be high; understanding this, clinicians might opt for a more aggressive dosing regimen (or a prolonged infusion) to ensure therapeutic levels are achieved.

The route of administration decisions can be informed by genetics. If a patient requires oral ketamine for chronic pain management, knowing that CYP3A4 plays a dominant role in first-pass metabolism is essential. Those on medications that induce CYP3A4 (or with high-expression genotypes) may experience lower oral ketamine bioavailability [21]; alternative routes (IV, subcutaneous, or intranasal) or using a CYP3A4 inhibitor adjunct could be considered to boost effect. In contrast, patients with genetic or drug-related CYP3A4 suppression could be at risk of enhanced oral ketamine exposure, so dose adjustments and avoidance of additional 3A4 inhibitors would be prudent. This kind of drug–gene interaction management is a key aspect of personalized ketamine therapy.

Incorporating COMT and pain susceptibility genes into the pre-treatment assessment may help tailor multimodal pain management. For an individual with genetic markers indicating a high risk of severe PLP (e.g., KCNS1 risk allele or COMT Met/Met implying high pain sensitivity) [36], one might lean towards early and aggressive interventions such as perioperative ketamine infusions to preempt central sensitization. Meanwhile, someone with a genotype profile suggesting lower pain amplification might be managed with more standard approaches and reserve ketamine for breakthrough pain or refractory cases. In essence, genomics can stratify patients by likely benefit: those predicted to have high neuropathic pain burden stand to gain the most from ketamine’s NMDA-receptor blockade.

Monitoring and supportive care can be individualized. If a patient is a NET T-allele carrier, extra precautions (continuous blood pressure monitoring, readiness to treat hypertension) should accompany ketamine infusions [34]. Knowledge of such a genotype in a cardiac-compromised amputee patient might even sway the clinician to avoid ketamine or use a very low dose, opting for alternative analgesics to avoid cardiovascular stress. This illustrates how pharmacogenomics can improve safety through proactive risk management.

It is worth noting that routine pharmacogenetic testing for ketamine use is not yet standard practice. However, as the evidence grows, institutions could implement panels that include CYP2B6 and other relevant genes for patients likely to receive ketamine (for pain or depression). In oncology and psychiatry, genotype-guided therapy is already used for certain drugs; pain management is following suit [36]. Even without formal testing, clinicians should be aware of these genetic factors. For example, unexplained strong reactions to ketamine (either lack of effect or excessive side effects) might prompt consideration of an underlying metabolic polymorphism. Integrating pharmacogenomics with clinical factors (age, liver function, concomitant drugs) leads to individualized ketamine therapy.

## 8. Limitations of Current Research

Despite growing evidence supporting the use of ketamine for PAP, significant limitations persist in the current research landscape. These gaps affect the strength of clinical recommendations and the future direction of personalized analgesia approaches.

First, most available studies are constrained by small sample sizes and heterogeneous patient populations. Trials often group different types of amputations, upper limb, lower limb, traumatic, and surgical, without stratification. This heterogeneity makes it difficult to determine ketamine’s specific efficacy for lower limb amputations, which uniquely affect mobility, balance, and weight-bearing ability, in contrast to the fine motor deficits associated with upper limb loss.

Second, there is substantial variability in dosing regimens, timing, and administration routes. Studies have employed IV, oral, epidural, and subcutaneous routes with differing dosage amounts and infusion durations. This inconsistency complicates direct comparisons across trials and obstructs efforts to develop standardized treatment protocols for perioperative and chronic PAP management [2,13].

Third, while ketamine has shown promise in reducing acute pain and possibly preventing chronic PLP, long-term outcome data remain limited. Few studies have followed patients beyond the immediate postoperative period to assess the durability of analgesia, incidence of chronic PAP, or neurocognitive side effects such as cognitive decline, which have been implicated in long-term ketamine use [46,47].

Fourth, pharmacogenomic factors influencing ketamine metabolism, efficacy, and tolerability are only beginning to be explored. Although variations in genes such as CYP2B6, COMT, and SLC6A2 have demonstrated potential relevance [22,32,34], no RCTs have yet incorporated genotype-guided ketamine dosing or stratified outcomes based on pharmacogenomic profiles. This remains a critical opportunity for advancing precision analgesia and further expanding the scope of treatment.

Finally, psychosocial variables and preoperative factors such as pre-existing limb pain, depression, and anxiety, which can significantly affect PAP outcomes, are often underreported or uncontrolled in existing studies, introducing additional bias.

Future research priorities should include conducting large-scale, amputation-type-specific RCTs, standardizing ketamine dosing, timing, and route protocols for PAP. Longitudinal studies are also needed to track long-term analgesic efficacy and neurocognitive safety. In addition, integrating pharmacogenomic testing into trial designs may facilitate more personalized therapy while also accounting for psychosocial and preoperative risk factors in outcome analyses. Addressing these limitations will be critical for establishing evidence-based, personalized ketamine strategies to improve pain control and quality of life for amputees.

## 9. Conclusions

Ketamine emerges as a valuable tool in the evolving management of PAP, offering mechanisms that directly target central sensitization, neuropathic pain pathways, and maladaptive neuroplasticity. Its use, particularly in perioperative settings, represents an opportunity not only to treat established pain but also to potentially prevent the development of chronic PLP. However, variability in clinical responses highlights the need for a more individualized approach rather than a uniform protocol.

Pharmacogenomics provides a critical path forward. Genetic variations in enzymes such as CYP2B6 and CYP3A4/5 and modulators of pain perception, like COMT and SLC6A2, influence ketamine’s pharmacokinetics, efficacy, and side effect profile. Integrating pharmacogenomic profiling into clinical practice could enable precision ketamine therapy—optimizing dosing, minimizing adverse events, and improving long-term outcomes for amputees.

Clinicians should begin to recognize that ketamine is not a “one-size-fits-all” therapy. Instead, pre-treatment risk stratification based on genetic, clinical, and psychosocial factors may guide the selection of candidates most likely to benefit from ketamine while mitigating potential harm in vulnerable individuals. Early adoption of genotype-informed protocols, even if initially limited to high-risk patients, could accelerate the clinical transition toward precision analgesia in amputation care.

Future research must prioritize large-scale RCTs and the integration of pharmacogenomic and biomarker analyses into trial design. Personalized pain medicine, once theoretical, is becoming a necessary evolution. Ketamine offers an early platform to demonstrate how precision analgesia can be realized in real-world clinical settings, fundamentally improving the quality of life for patients facing the profound consequences of limb loss.

## Figures and Tables

**Table 1:** Summary of ketamine administration regimens for post-amputation pain management across clinical studies. Legend: PAP—post-amputation pain, PLP—phantom limb pain, IV—intravenous, AKA—Above-Knee Amputation, BKA—Below-Knee Amputation, TID—Three Times Daily, POD—Postoperative Day.

Author(s)	Route of Administration	Timing	Dose	Duration	
Nikolajsen et al. [3]	IV	Postoperative (PLP present)	Bolus 0.1 mg/kg over 5 min, then infusion 7 mcg/kg/min for up to 45 min	Up to 45 min	n = 11; uncontrolled; short relief only.
Wilson et al. [4]	Epidural	Perioperative	Bolus 0.5 mg/kg, infusion 3.3 mg/kg/L at 15 mL/h, post-op 10–20 mL/h	48–72 h	RCT; small sample; significant pain reduction.
Hayes et al. [5]	IV	Perioperative	Bolus 0.5 mg/kg, infusion 0.15 mg/kg/h	72 h	RCT; underpowered; no significant benefit.
Eichenberger et al. [6]	IV	Postoperative (chronic PLP)	Not fully specified	48 h	Moderate sample; partial blinding; combo with calcitonin significant.
Polomano et al. [7]	IV	Acute trauma recovery (combat-related)	120 mcg/kg/h for most; two patients received 60 or 100 mcg/kg/h	3 days	n = 19; non-randomized; benefit in subset only.
Buvanendran et al. [8]	Oral	Pre- and postoperative	1 mg/kg 1h pre-op, repeated 8h later; 1 mg/kg TID POD1, 0.5 mg/kg TID POD2	2 days	Case series (n = 3); low quality; reported benefit.

**Table 2:** Key pharmacogenomic factors in ketamine therapy.

Gene (Variant)	Effect on Ketamine PK/PD	Clinical Impact	References
CYP2B6 (*6 allele)	↓ Enzyme activity → slower N-demethylation of ketamine. Higher ketamine/norketamine ratio (accumulation).	↑ Ketamine levels: prolonged effect. More dissociative side effects were observed in *6 carriers. Consider dose reduction.	[22]
BDNF (Val66Met)	Val66Met polymorphism → ↓ activity-dependent BDNF secretion → impaired neuroplasticity. No direct effect on ketamine metabolism, but may modulate response via synaptic plasticity.	May reduce response to ketamine in CNS conditions (e.g., depression, potentially chronic pain). In pain, impaired neuroplasticity may contribute to persistent central sensitization and reduced ketamine efficacy, especially in PLP. Interaction with CYP2B6 and COMT variants may affect tolerability and dosing.	[25,29,30,31]
CYP3A4/5 (*22 or *1)	CYP3A4: major contributor, especially orally. 22 allele = ↓CYP3A4; CYP3A5*1 = additional metabolism.	Oral ketamine: high first-pass metabolism (CYP3A4) polymorphism or inhibitors cause significant PK changes. IV: CYP3A4 vs. CYP2B6 balance affects clearance. Monitor for drug interactions.	[22]
COMT (Val158Met)	Met allele → ~75% ↓COMT activity → ↑ dopamine/NE levels in CNS. It does not metabolize ketamine but alters pain modulation.	Low COMT activity is associated with ↑ pain sensitivity and variable opioid requirements. It may influence ketamine analgesic needs (e.g., high-pain-sensitivity patients might require higher or prolonged dosing; still under study).	[31,32]
SLC6A2 (NET rs28386840)	T allele → ↓ NET expression → impaired NE reuptake. No direct effect on ketamine metabolism.	Exaggerated cardiovascular response: T carriers have faster, higher blood pressure rise on ketamine. Higher risk of acute hypertension necessitates close monitoring or dose caution.	[34]
KCNS1 (Ile/Val SNP)	Polymorphism in Kv9.1 potassium channel; no effect on ketamine PK.	Val variant linked to a higher incidence of PLP. Identifies patients with severe pain phenotype who may particularly benefit from NMDA blockade (theoretical use in preemptive ketamine analgesia).	[36]

**Table 3:** Comparison of ketamine and other treatments for post-amputation pain: mechanisms, effectiveness, side effects, and limitations.

Treatment	Mechanism	Duration	Effectiveness in PAP	Side Effects	Limitations	Primary or Adjunct Use	References
Ketamine	NMDA receptor antagonist, reduces central sensitization and neuroinflammation	Short-term to long-term effects possible	High efficacy in refractory cases, perioperative administration reduces long-term pain	Neuropsychiatric effects (hallucinations, dissociation), hypertension, nausea	Neuropsychiatric side effects, unclear long-term safety, lack of standardized protocols	Adjunct, potentially primary in refractory cases	[2,13,37]
Opioids	Binds to opioid receptors, inhibits pain transmission	Short-term (hours)	Effective for acute pain but limited for chronic PAP	Addiction risk, tolerance, opioid-induced hyperalgesia, respiratory depression	High addiction potential, limited chronic pain efficacy, cognitive effects	Primary for acute pain, adjunct for chronic	[2,38,39]
Antidepressants (TCAs, SNRIs)	Enhances serotonin and norepinephrine inhibition of pain pathways	Long-term if effective	Limited evidence, may help with associated depression	Sedation, dry mouth, dizziness, cardiac arrhythmias	Delayed onset, high side effect burden, weak evidence for PAP	Adjunct	[38,40,41]
Anticonvulsants (Gabapentin, Pregabalin)	Modulates calcium channels to reduce excitatory neurotransmission	Long-term	Mixed results, may help in neuropathic components	Dizziness, peripheral edema, renal impairment, drowsiness	Need for titration, mixed results for PAP	Adjunct	[2,38,39]
Local Anesthetics	Blocks sodium channels to prevent pain signal transmission	Short-term (hours)	Useful for acute pain but requires repeat administration	Motor weakness, cardiotoxicity with bupivacaine	Short duration, limited efficacy in neuropathic pain	Adjunct	[41,42,43]
NMDA Antagonists (Memantine, Dextromethorphan)	NMDA receptor antagonists but weaker effects than ketamine	Short-term to none	Weak or inconsistent efficacy	Dizziness, nausea, agitation, headache	Weak NMDA antagonism, inconsistent results	Adjunct	[2,41,44]
Calcitonin	Modulates neurogenic inflammation and neuronal firing	Short-term	Some benefit, particularly in combination with ketamine	Flushing, hypocalcemia, dizziness, nausea	Limited data, side effects reduce tolerability	Adjunct	[2,38,41]
Non-Pharmacological Therapies	Includes mirror therapy, CBT, spinal cord stimulation, TENS	Varies	Effective for some, especially mirror therapy and spinal cord stimulation	None for psychological therapies, device-related risks for TENS, SCS	Variable effectiveness, requires adherence and training	Adjunct	[2,39,45]

## Data Availability

The original contributions presented in this study are included in the article. Further inquiries can be directed to the corresponding author.

## Abbreviations

The following abbreviations are used in this manuscript:

AKA	Above-Knee Amputation
AMPA	α-Amino-3-hydroxy-5-methyl-4-isoxazolepropionic Acid
BDNF	Brain-Derived Neurotrophic Factor
BKA	Below-Knee Amputation
CBT	Cognitive Behavioral Therapy
CNS	Central Nervous System
COMT	Catechol-O-Methyltransferase
CYP	Cytochrome P450
DNA	Deoxyribonucleic Acid
FDA	Food and Drug Administration
GABA	Gamma-Aminobutyric Acid
GABAa	Ionotropic GABA Receptor
GRIN2B	Glutamate Ionotropic Receptor NMDA Type Subunit 2B
H_2_O_2_	Hydrogen Peroxide
IV	Intravenous
KCNS1	Potassium Voltage-Gated Channel Subfamily S Member 1
Met	Methionine Allele (in Polymorphisms)
mTOR	Mechanistic Target of Rapamycin
NE	Norepinephrine
NET	Norepinephrine Transporter
NMDA	N-Methyl-D-Aspartate
PAP	Post-Amputation Pain
PK/PD	Pharmacokinetics/Pharmacodynamics
PLP	Phantom Limb Pain
POD	Postoperative Day
PPI	Present Pain Intensity
RCT	Randomized Controlled Trial
ROS	Reactive Oxygen Species
RLP	Residual Limb Pain
SCS	Spinal Cord Stimulation
SLC6A2	Solute Carrier Family 6 Member 2
SNRI	Serotonin-Norepinephrine Reuptake Inhibitor
SSRI	Selective Serotonin Reuptake Inhibitor
TCAs	Tricyclic Antidepressants
TENS	Transcutaneous Electrical Nerve Stimulation
TrkB	Tropomyosin Receptor Kinase B
Val	Valine Allele (in Polymorphisms)
